# From Film to Data: Automating Meta-Feature Extraction in Historical Aerial Imagery

**DOI:** 10.1007/s41064-025-00357-8

**Published:** 2025-09-03

**Authors:** Felix Dahle, Yushan Liu, Roderik Lindenbergh, Bert Wouters

**Affiliations:** 1 Department of Geoscience and Remote Sensing, TU Delft, Delft, The Netherlands; 2 Department of Control and Operations, TU Delft, Delft, The Netherlands

**Keywords:** Historical imagery, Fiducial marks, Computer vision, Altimeter, Meta-data

## Abstract

Historical aerial imagery provides valuable data from regions and periods with limited geospatial information. A common method to utilize this data is through the generation of ortho-photos and 3D models using Structure-from-Motion (SfM) techniques. However, many of these images were scanned decades after their acquisition and require geometric calibration, along with internal and external camera parameter estimation, for accurate reconstruction. Manual identification of key features, such as fiducial marks and text annotations, is labour-intensive, while existing automated methods struggle with poor-quality datasets.

This paper presents an automated workflow that combines computer vision and machine learning techniques to detect and extract these key features from historical aerial images. To address challenges related to image quality, we also introduce estimation protocols that compensate for missing or unreliable detections by leveraging redundancy across multiple flight paths. The methodology was evaluated on the TMA (Trimetrogon Aerial) archive, a collection of historical images from the Antarctic Peninsula. Our test dataset comprised over 7000 images from 20 different flight paths. The workflow demonstrated high success rates in detecting and extracting fiducial marks, image subsets, and textual annotations. Approximately 70% of the images provided usable focal length data, while fiducial mark detection exhibited high accuracy except in cases of severe scanning artifacts. Altitude data extraction proved to be the most challenging, with successful results in only 15% of images due to degraded altimeter readings. Despite these limitations, the automated workflow effectively estimated missing parameters, ensuring robust image reconstruction across flight paths. The code for this workflow is open-source and publicly available on GitHub at https://github.com/fdahle/hist_meta_extraction.

## Introduction

Historical aerial images are a valuable source of information for a wide range of applications, from environmental (Heisig and Simmen 2021), cryospheric (Pope et al. 2014) to archaeological sciences (Cowley and Stichelbaut 2012), but still remain under-exploited (Kostrzewa 2024). A particularly prominent application involves generating ortho-photos and 3D models through Structure-from-Motion (SfM) techniques (Farella et al. 2022; Mestre-Runge et al. 2023; North and Barrows 2024). While SfM can estimate camera parameters from image data alone, its accuracy and reliability are significantly improved when internal parameters (e.g., focal length) and external information (e.g., camera height or position) are known (Stark et al. 2022; Zhang et al. 2021). Many of these images are scanned with photogrammetric scanners at high resolution (Kostrzewa et al. 2024); however, the scanning process itself often introduces subtle distortions, which can affect the overall consistency of the dataset (Schulz et al. 2021). Therefore, to perform reliable geospatial analysis, scanned images must be normalized by calibrating their geometry based on fiducial marks.

Fiducial marks are reference points embedded in the original film to correct geometric distortions. While their film-space coordinates are typically provided in camera calibration reports, the corresponding image-space coordinates must be identified on each scanned image. Manual identification of fiducial marks is straightforward but time-consuming and labour-intensive. Unfortunately, in many cases, the original camera calibration reports have been lost over time, leaving only the images themselves. Additionally, scanned images often contain distortions, such as stretching and skewing, introduced by the scanning process, further complicating their use in precise geospatial applications.

Several automated methods have been proposed to address these challenges. Early research by (Sun and Wu 2001) employed attribute-based mathematical morphology to segment fiducial marks. Later, (Ye et al. 2016) introduced a semi-automatic approach based on circle fitting, specifically tailored for ARGON satellite imagery (Girod et al. 2018). proposed a shape-based method that identifies bright circular blobs near the image frame as proxies for fiducial mark locations. Other approaches, such as those by (Knuth et al. 2023) and (Salach 2017), rely on template matching to detect fiducial marks across image sets. These open-source methods vary in robustness and typically require manual correction, especially when applied to degraded or low-quality scans. Commercial software packages, including for example Agisoft Metashape (Agisoft o.J.), ArcGis Pro (Esri o.J.), or Trimble Inpho (Trimble o.J.), offer powerful and largely automated tools for fiducial mark detection that typically perform well, even on challenging imagery. However, these tools are paid and closed-source, which limits accessibility for some users. Their proprietary nature also reduces transparency, as internal algorithms are not openly documented or modifiable. This makes it difficult to adapt or extend their functionality, and complicates integration into fully automated and reproducible processing workflows.

**Fig. 1:**
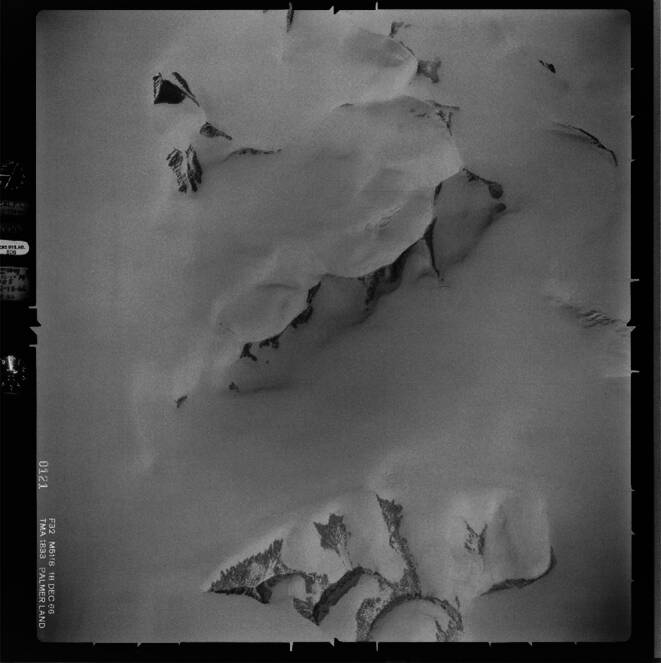
Example image CA183332V0121 from the TMA dataset

In this study, we utilize the Trimetrogon Aerial (TMA) archive, a collection of approximately 330,000 black-and-white aerial photographs captured by the U.S. Navy between 1946 and 2000. Around 2012, the imagery has been scanned at a resolution of 25 microns (1000 dpi) by the U.S. Geological Survey (USGS) and the Antarctic Geospatial Information Center (University of Minnesota) and is publicly available at (Polar Geospatial Center 2023). An example image from the archive is shown in Fig. 1. The focal lengths in this dataset range from 151.09 to 156.00 mm, while altitudes at the time of image acquisition vary between 8000 and 24,000 ft, depending on flight line and location. Notably, many images lack accompanying metadata, and approximate values for focal length or altitude are often unavailable.

Our ultimate goal is to extract three-dimensional information from these images using SfM. While in principle, internal and external camera parameters can be estimated during bundle adjustment (Mölg and Bolch 2017), the quality and reliability of the resulting 3D models can benefit from some prior knowledge, especially in the case of archival aerial imagery (AliAkbarpour et al. 2015; Knuth et al. 2023; Sevara et al. 2018). Historical datasets like TMA often have near-parallel viewing geometries and limited image overlap (around 60%), which can lead to unstable or degenerate configurations with inaccurate approximated camera parameters. This challenge is further increased by strong variations in image quality, caused by improper storage, exposure issues during acquisition, and distortions introduced during scanning, as seen in (Maiwald et al. 2023).

Given the large volume of images, a key objective was to automate the processing as much as possible for all parameters located in the images. Therefore, we have developed a fully automated workflow for detecting fiducial marks directly from the images. Our approach emphasizes robustness, allowing to estimate positions even when detection fails in degraded or incorrect scanned images. In addition to fiducial mark detection, our workflow enhances the geospatial use of the images by extracting height information from three-pointer altimeters embedded in each image. Such altimeters indicate altitude using three separate hands, providing vertical data that can improve the accuracy of 3D reconstructions. Finally, we extract metadata such as focal length from typewritten annotations found on the images.

This paper presents our methodology for the automatic extraction of fiducial marks and height information, with a particular focus on the TMA archive. However, our workflow is designed to be adaptable and can be applied to other historical aerial image collections. To the best of our knowledge, no previous study has developed an automated approach for extracting height data from three-pointer altimeters in historical imagery. By addressing this gap, our work offers an important next step to the challenges of utilizing historical imagery for geospatial analysis and 3D modelling.

## Methodology

The main objective is recovering metadata from scanned photos, whenever available. This metadata notably includes focal length and image altitude, which can be estimated through text extraction. Altitude information can also be derived from embedded altimeter readings. Another goal is to identify fiducial marks, which enable precise geometric calibration. These tasks share similar techniques but are designed to function independently. However, the inconsistent quality of the historical images poses challenges, and the methods may not always yield reliable results. To mitigate this, an automatic estimation step follows each extraction, serving as a fallback to ensure robustness and continuity in subsequent processing stages. A detailed description of these metadata recovery steps is presented in the following subsections.

### Text Extraction

Historical aerial images often contain hand-written or typewritten annotations directly on the photographs, such as focal length or altitude values. These annotations are both valuable and problematic: while they provide crucial metadata, they also introduce noise that can hinder automated processing. To address this, we employ text recognition software to both extract relevant information and mask the regions containing text. Traditional optical character recognition (OCR) methods, such as those based on TensorFlow, were initially considered but yielded poor results in our early trials, particularly when applied to typewritten annotations in historical imagery. While no systematic benchmark was performed, we observed that these general-purpose models, typically trained on modern machine-printed text, struggled with the lower contrast and variability of historical scans. In contrast, the OCR tool PP-OCR (Du et al. 2020), originally developed for recognizing Chinese characters, demonstrated significantly more robust performance in our use case. It not only recognized typewritten annotations more reliably but also provided bounding boxes for each detected text region, enabling downstream filtering and spatial reasoning.

Given the large size of the images, applying text recognition to the entire image in one pass is computationally impractical. Instead, we focus on the four border regions (N, E, S, W) where annotations are typically found. Each border is divided into overlapping sub-regions, and text extraction using PP-OCR is performed on each sub-region individually. To account for annotations that may be written upside down, the process is repeated with a 180-degree image rotation. This approach is sufficient for the current dataset, where text annotations are only present along the top and bottom edges. However, for other datasets where text may also appear along the sides, additional rotations—such as 90 and 270 degrees—can be included with minimal computational overhead. The extracted text and bounding boxes from all sub-regions are then merged, aligning overlapping text based on matching content at the start and end of adjacent regions. Finally, the separate bounding boxes are combined into a single comprehensive bounding box for each text region. This combined text data and bounding box information serves as input for further processing steps, such as masking text regions to reduce noise in subsequent analyses.

**Fig. 2:**
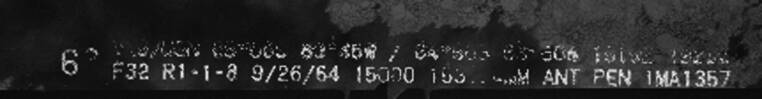
Example text from an image from the archive. Note that height (15,000) and focal length (153.xx mm) is included in the description

Despite successful localization of the text regions, extraction of useful information remains a challenge due to incomplete detections and false positives, as illustrated in Fig. 2, where parts of the focal length annotation are obscured. To improve reliability, we leverage prior domain knowledge and statistical redundancy across all images from the same flight path and camera orientation. For each image, all detected text elements are concatenated into a single string and stored for analysis. We then apply regular expressions to identify substrings that likely represent focal lengths or altitudes: values containing a decimal point or the unit “mm” for focal lengths, and numerical patterns ending in “000” for altitudes. The identified strings are standardized to a fixed format, such as a 7-character focal length (e.g., XXX.XX) or a numeric altitude value, and recorded per image as a text string in a database. Unrecognized characters are replaced with a placeholder (e.g., “X”) to mark uncertainty. To enhance reliability, all extracted values from a given flight path are compared character by character to determine the most frequently occurring digit or symbol at each position. For example, if some images yield 15X.3X mm and others give 154.X4 mm, the inferred result may be 154.34 mm. This voting-based approach helps correcting OCR errors and fill in uncertain or missing characters. Since the OCR does not provide per-character confidence scores, we treat any non-numeric characters in focal length fields as invalid and exclude them from voting. Finally, we incorporate domain-specific constraints, for instance, focal lengths in this archive are always above 150 mm, so any extracted value like for example 54.34 mm is automatically adjusted to 154.34. This structured and knowledge-guided approach enhances metadata extraction by reducing sensitivity to single-image OCR errors and leveraging the redundancy of similar images within a flight line.

### Fiducial Mark Detection

**Fig. 3:**
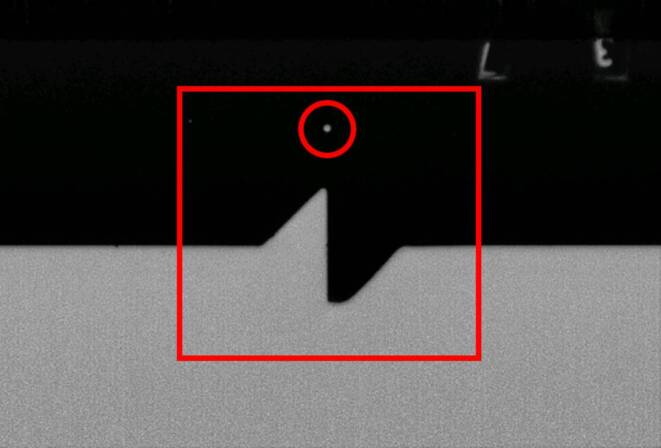
Example for a fiducial pattern (red box) together with the fiducial mark (red circle)

Figure 3 illustrates how we identify fiducial marks on historical images. A fiducial mark is a precise reference point, often a small dot, crucial for image orientation and calibration. Surrounding this mark is a distinctive fiducial pattern, which aids in accurately locating the mark. The pattern is designed with lines converging precisely on the fiducial mark to ensure accurate detection.

**Fig. 4:**
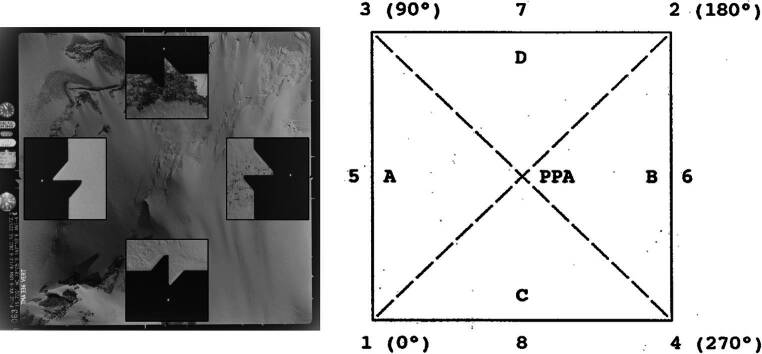
Example for fiducial marks in the dataset and the equivalent schematics from a camera calibration report

As shown in Fig. 4, the fiducial marks are located at eight positions. However, only four of these marks (5–8) can be directly detected, while the remaining four (1–4) must be inferred. Once the positions of the first four fiducial marks are established, the principal point of autocollimation (PPA) (McGlone et al. 2013), can be estimated as the point of intersection of the lines connecting them.

**Fig. 5:**
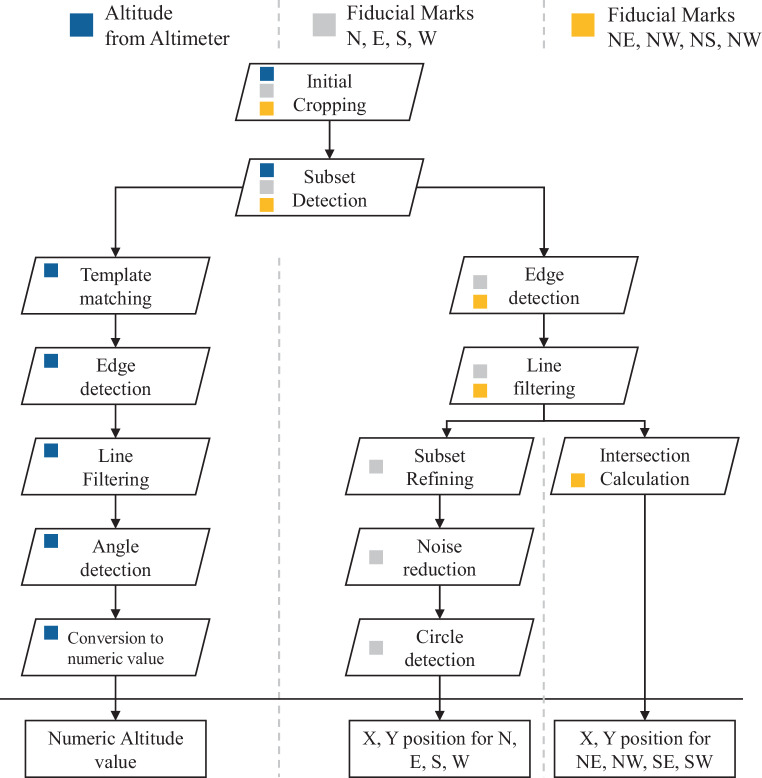
Steps required for obtaining altitude, fiducial marks for N, E, S, W and fiducial marks for NE, NW, NS, NW. Each step is colour coded to show which steps are required for each extraction

To detect fiducial marks automatically, we employ a structured seven-step approach as shown in Fig. 5, applied separately for each fiducial mark. For the cardinal directions (North, East, South, West), direct detection is possible, while for the intercardinal directions (Northeast, Southeast, Southwest, Northwest), fiducial marks must be computed based on detected cardinal marks.

#### Fiducial Marks for N, E, S, W

**Fig. 6:**
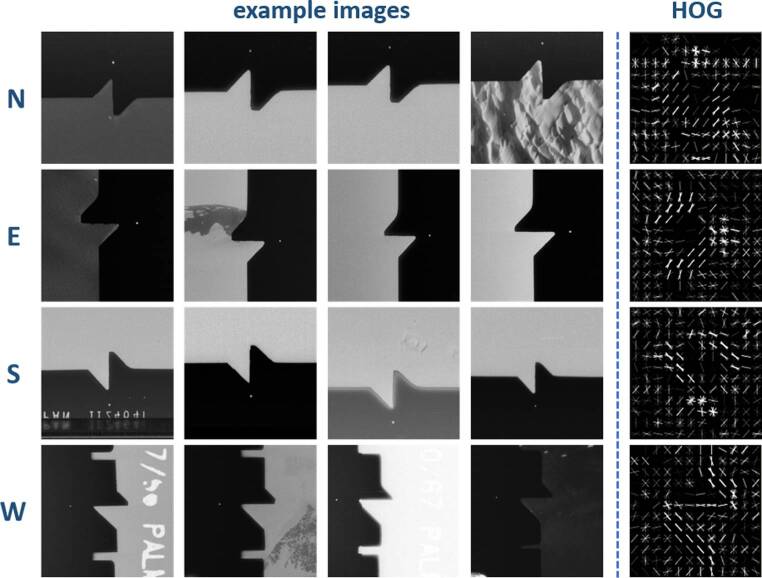
Example for fiducial marks from different images and their HOG filters for each cardinal direction N, E, S, W

Fiducial marks in the four main directions are detected through a machine-learning and computer vision based approach, consisting of the following steps:

1. Initial Cropping: Since fiducial marks are always positioned near the image borders, we extract a cropped region at each respective boundary. This preliminary step enhances processing speed and accuracy by reducing the search space for the fiducial pattern.
2. Subset Detection: Once the cropped region has been extracted, we use dlib, a versatile toolkit designed for machine learning and computer vision (King 2009), to recognize the fiducial patterns in that region. We employ the so-called simple object detector to detect fiducial patterns in the images. This efficient object detector is a trainable model based on Histogram of Oriented Gradients (HOG) and a linear Support Vector Machine (SVM) classifier. To enable the detection, we created a training set of 100 fiducial patterns for each direction, enabling the model to learn and generalize effectively. The HOG technique effectively extracts dominant image gradients, which in our case point towards the fiducial marks. Extracted HOG gradients and corresponding fiducial patterns can be seen in Fig. 6.

**Fig. 7:**
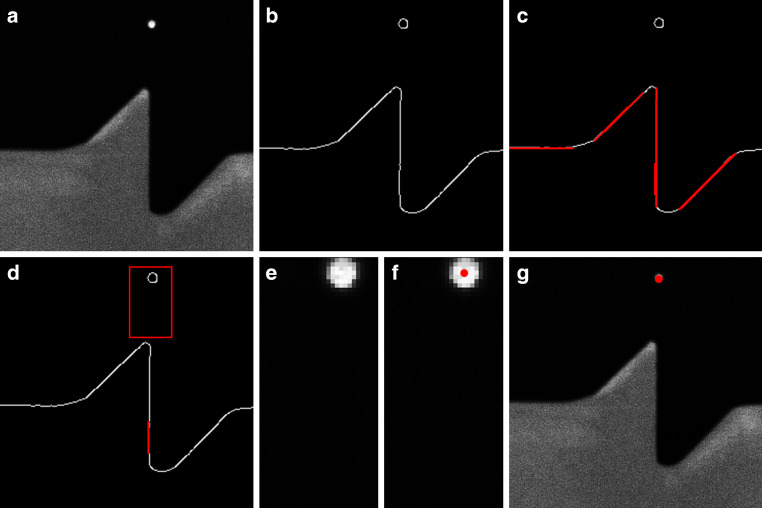
Steps for fiducial mark extraction: detected subset (**a**), binarization (**b**), Edge detection (**c**), filtering for centre line (**d**), blurred subset (**e**), subset with detected point (**f**), final absolute coordinates of subset (**g**)

At this stage, precise localization is not necessary; rather, we aim to isolate a smaller region as a subset for further refinement by only looking at the border regions of an image. If no suitable subset is found, or if more than one subset is detected, detection for this direction is halted. Figure 7 is showing different stages of this extraction in panels A‑G.

3. Pre-processing: To simplify subsequent operations, the subset (panel A) is blurred and then binarized using a dynamic threshold (resulting in panel B). Instead of a fixed threshold, we compute the most frequent pixel value (typically the background) and adjust the threshold dynamically by adding a safety margin of 10 px.
4. Edge Detection and Line Filtering: Canny edge detection, (Xu et al. 2017), is applied to extract straight lines in the subset (panel C), which serve as structural references for fiducial mark localization. Only lines with the correct orientation (vertical for N/S, horizontal for E/W) are retained. Among these, the line closest to the centre is selected (panel D). If multiple lines are detected, they are merged by averaging their endpoints.
5. Refining the Region of Interest: Using the detected line as a guide, we extract a smaller subset (panel E), expanding the region along the fiducial direction while narrowing it perpendicularly.
6. Noise Reduction and Circle Detection: At high zoom levels, image artifacts are common. To mitigate this, we apply the morphological operations erosion and dilation to remove small artifacts while preserving key structures. We then search for circular features within a predefined size range using the circle Hough transform. As a fallback, we detect image moments, which can also identify non-perfect shapes (e.g., blurry or slightly oval ones), but only with pixel-level accuracy. Image moments are weighted averages of image intensities that describe the shape’s geometry—such as its centre of mass, orientation, or area—and are useful for locating and characterizing objects when perfect shapes are not present. If multiple circles are detected, the one nearest to the centre is selected (panel F) If no circle is found, the subset is gradually shifted outward until a fiducial mark is detected or the image boundary is reached. If no mark is found, detection for this direction remains incomplete.
7. Final Localization: Once the position of the fiducial mark is determined within the subset, its absolute coordinates in the full image are calculated (panel G).

#### Fiducial Marks for NE, SE, SW, NW

The remaining four fiducial marks cannot be directly detected, as they lack distinct white dots marking their positions. Instead, their locations are inferred based on the detected cardinal fiducial marks. This process involves the following steps:

1. Resizing Subsets for Edge Detection: Prior to performing Canny edge detection, the relevant subsets from the adjacent cardinal fiducial marks (e.g., North and East for the Northeast fiducial mark) are resized. The subset is elongated in the primary direction of interest while reducing its width in the perpendicular direction.
2. Edge Detection and Line Extraction: Canny edge detection is applied to identify lines perpendicular to the expected fiducial mark location. Only the lines closest to the centre of the subset are retained. If multiple lines are detected due to interruptions in the fiducial pattern, they are averaged to form a single representative line.
3. Intersection Calculation: The detected lines from the two relevant subsets (e.g., North and East for the Northeast fiducial mark) are used to calculate their intersection point. This intersection is assigned as the inferred fiducial mark position.

By combining direct detection for cardinal fiducial marks with computed positions for intercardinal marks, this approach enables robust and accurate fiducial mark localization across historical aerial images. This methodology ensures consistency across the dataset while accounting for variations in image quality, exposure, and potential distortions.

### Height Detection

**Fig. 8:**
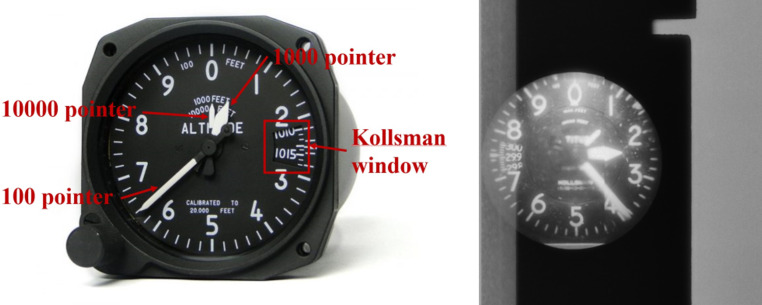
Example for a physical altimeter and it’s equivalent from a historical image of the TMA archive. Adapted from

Height detection involves extracting the altitude data from either text annotations or an altimeter display in the images. In some cases, height is clearly visible as text, which can be processed as described in section Sect. 2.1. However, in many cases, text annotations are absent, and the height must instead be derived from a three-pointer altimeter, as shown in Fig. 8. This altimeter measures altitude through air pressure and displays it using three clock-like pointers: the longest pointer indicates increments of 100 feet, the next pointer 1000 feet, and the shortest pointer 10,000 feet. Similar to fiducial mark detection, height detection required a multi-step approach, as depicted at the left side of the workflow in Fig. 5.

The first step in this process is to localize the altimeter within the image. Since it is typically found at the bottom-left corner and has a distinct round shape with labelled numbers, its position is easy to approximate. We use again the object detection framework of dlib, trained on 100 manually annotated images with various lighting conditions and image qualities. This small training set suffices due to the standardized design of the altimeter across the dataset.

Next, template matching is applied to refine the position. Perfect scans would allow circle detection through methods like Hough transforms, but the digitization of these historical images often results in slight distortions. To address this, template matching focuses on the detection of numeric labels such as 3, 5, and 8, which are consistently present on the altimeter face. This approach improves robustness, even in cases where parts of the altimeter are cut off.

**Fig. 9:**
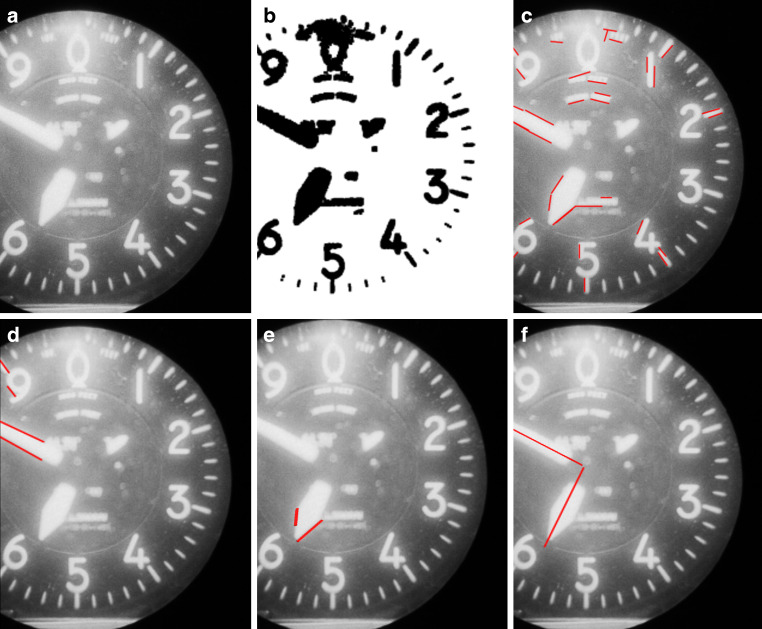
Steps for altitude extraction using an altimeter: Original image (**a**), binarization (**b**), Edge detection (**c**), filtering for centre lines (**d**), filtering for tip lines (**e**), final lines (**f**)

Once the position of the altimeter is identified, image enhancement is performed to compensate for inconsistencies in brightness and clarity caused by reflections or poor lighting. We apply histogram equalization to the central area of the altimeter, which improves contrast and prepares the image for binarization. The outer areas, where reflections are more prominent, are set to the median pixel value of the circular region to avoid interference (Step A in Fig. 9).

After enhancement, edge detection is carried out on the binarized image (Step B) using the Canny method to extract lines within the altimeter (Step C). Only lines oriented towards the circle’s centre are retained, and additional filtering is applied to distinguish each pointer based on its characteristics. The 100 feet pointer is recognized by long, parallel lines (Step D), while the 1000 feet pointer has intersecting tip lines (Step E).

With the relevant lines detected, we calculate the angle of each pointer relative to the zero position on the altimeter. The angles range from 0 to 360°, where 0° corresponds to position zero and 180° to position five. From these angles, the readings of the 100 and 1000 feet pointers are determined directly.

Detecting the 10,000 feet pointer presents a unique challenge. Its small size and placement near the bottom of the altimeter often result in poor visibility. However, since flight heights rarely exceed 30,000 feet in the dataset, the pointer’s position will always fall within the first few digits (0 to 3). Therefore we calculate three possible positions for the 10,000 feet pointer and search for it within these areas. If detected, this final step confirms the complete altitude reading.

By analysing the positions of all pointers relative to the instrument’s centre, the altitude can be converted into a numeric value. The angles of these pointers are measured relative to the north direction and mapped onto a circular scale, where a full rotation corresponds to 10,000 feet for the shortest pointer, 1000 feet for the middle pointer, and 100 feet for the longest pointer. The final altitude is obtained by summing these values. However, if the calculated altitude falls below 8000 feet, an offset of 10,000 feet is added, as for the considered dataset no planes flew below this altitude. This correction accounts for cases where the 10,000-feet pointer was not detected correctly. In some cases pointers can overlap, which happens most frequently with the 10,000-feet pointer. In these instances, the algorithm handles the situation similarly to cases when the 10,000-feet pointer could not be detected. Overlaps between the 100-feet and 1000-feet pointers are very rare and were therefore not explicitly addressed.

### Estimation & Quality Control

Detection of fiducial marks and altimeter readings is not always successful. Low contrast, excessive image artifacts, or incorrect scanning can hinder automated recognition. In some cases, fiducial marks or altimeter displays may have been cut off partially or entirely during the scanning process.

To handle these issues, we apply a correction strategy based on redundancy of parameters within the same flight line. Images captured along a flight path typically share the same camera setup, calibration parameters, and flight altitude, which allows for cross-validation between overlapping observations. On average, each flight path contains approximately 54 images, providing in most cases sufficient redundancy for correction. A value is flagged as missing if no meaningful number could be extracted, and it is marked as an outlier if it deviates by more than two standard deviations from the median of all valid values in the same sequence. Only flight paths with at least three valid (non-null) entries are considered for correction.

For the fiducial mark detection, if the initial subset containing the mark is not detected, we first estimate its likely position based on the average coordinates of subsets detected in all other images from the same sequence. Once this position is determined, the fiducial mark detection algorithm is re-applied to the estimated region. If the extraction is still unsuccessful, we calculate the final coordinates of the missing fiducial mark by averaging the coordinates of matching marks from other images of the same flight path.

A similar procedure is applied for altimeter readings. If the altimeter display is not detected in an image, we begin by estimating its expected position. If the reading remains missing, the altitude value is inferred by analysing the heights recorded in other images within the same flight line. Since the aircraft is assumed to fly along a stable trajectory, any obvious outliers are removed using the interquartile range (IQR) method. A weighted average of the remaining altitude values is then calculated to estimate the missing height. While this estimation is generally less precise than that for fiducial marks due to potential fluctuations in altitude, it provides a reasonable approximation that supports subsequent processing steps.

## Results & Discussion

To evaluate our methodology, we selected the 20 longest flight paths^1^ from the Antarctic Peninsula section of the TMA archive. This selection of 7719 images provides a diverse range of examples to thoroughly test our approach while still allowing us to apply and validate our estimation techniques across a large dataset. While this facilitates testing the full potential of our method, it may lead to slightly higher estimation success rates compared to shorter or less continuous flight paths found elsewhere in the archive.

Table 1 provides an overview of the test results from 20 flight paths. It summarizes the extraction process for each key feature, including subsets used as a preliminary step in fiducial mark detection. The table shows the number of entries that were successfully extracted automatically, those estimated based on extracted features from other photos from the same flight path, and those that remained missing due to failed extraction or estimation. The final column presents the results of a manual extraction of the computer vision based parameters, indicating whether a human observer could identify these patterns on the images. This serves as a baseline for comparison with the automatic extraction, with the exact values per flight path shown in the Appendix.

**Table 1:** Summary of extracted, estimated, missing and possible manual values for text extraction, fiducial mark detection and height detection for 7719 photos from 20 different flight paths. (the values with n.e. were not considered in the manual extraction).

Field	Extracted	Estimated	Missing	Manual
Text position	7719	0	0	7719
Focal length	2145	3436	2138	n.e.
Subset N	5791	1706	222	n.e.
Subset E	6828	779	112	n.e.
Subset S	6813	794	112	n.e.
Subset W	6859	748	112	n.e.
Fid mark 1 (SW)	7064	543	112	n.e.
Fid mark 2 (NE)	6990	508	222	n.e.
Fid mark 3 (NW)	6950	529	239	n.e.
Fid mark 4 (SE)	7150	457	112	n.e.
Fid mark 5 (W)	6552	1055	112	7711
Fid mark 6 (E)	6858	749	112	7714
Fid mark 7 (N)	5192	1475	1054	6015
Fid mark 8 (S)	6488	1119	112	7662
Height (text)	12	312	7395	n.e.
Height (altimeter)	1279	6440	0	5237

**Fig. 10:**
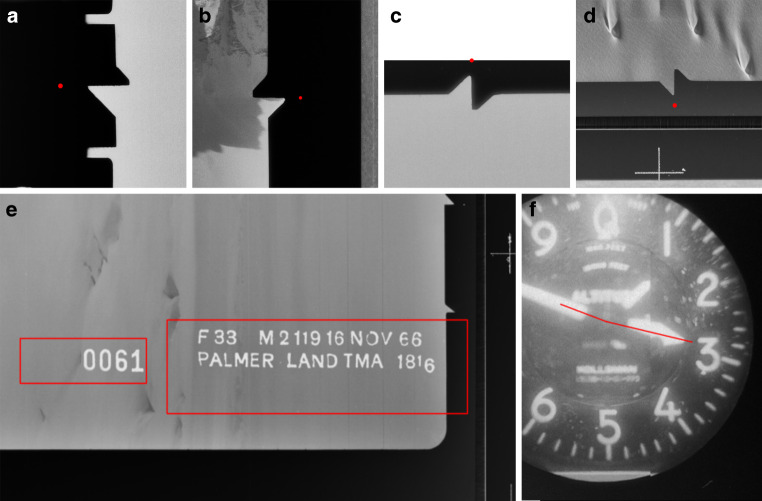
Examples for successful detections: Fiducial marks (top), text extraction (bottom left) and altitude based on altimeter (bottom right)

Figure 10 illustrates examples of successful data extraction. For each identified text, bounding boxes are available to determine the position of the text box. In the case of fiducial marks, precise coordinates are marked at the exact location of each fiducial spot. For height extraction using altimeters, three lines extend from the centre of the circular display to indicate the direction and position of the pointers. The altitude value of these pointers corresponds to 22,800 feet.

The results of the data extraction process demonstrate a high level of effectiveness in several areas. Text extraction performs exceptionally well, with no missing text boxes across the dataset. As can be seen in Table 1, for approximately 70% (5581 individual photos) of the images we could successfully extract focal length values. For the remaining 30% of the images, extraction of focal length data failed due to two primary factors. First, the complete absence of focal length information in the text. Second, poor legibility of the written text, as illustrated in Fig. 2, where the focal length is only partially discernible.

Subset extraction is similarly reliable, with only a small number of images missing critical data. Failures in this area are primarily the result of either portions of the image being cut off during scanning or images being scanned in an inverted orientation, which disrupts the fiducial pattern detection with dlib. This notably affects the left looking direction of flight path 1822, which explains the identical number of missing values for the subsets. Nevertheless, the overall success rate is sufficient to allow substitution of missing subsets with data from successfully processed images.

**Fig. 11:**
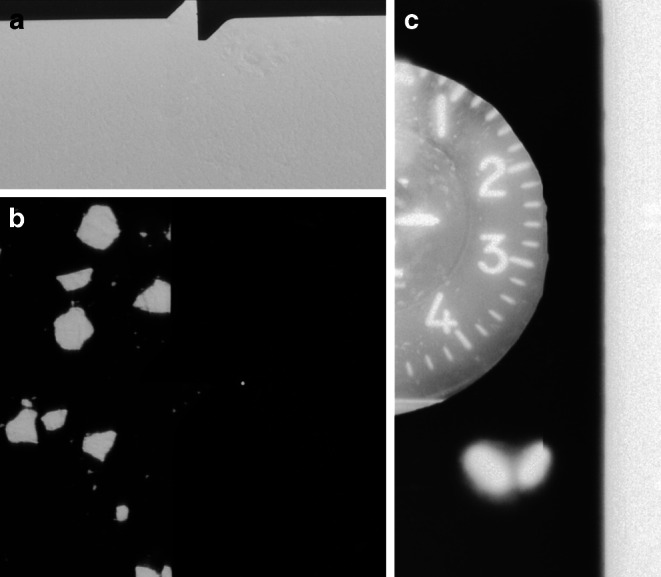
Examples for failed extractions: Fiducial mark detection with cut-off or difficult data (**a**, **b**) and height detection (**c**) with missing pointers

Fiducial mark extraction performs well overall but faces specific difficulties with fiducial mark 7, located at the top of the image. Investigations revealed that many of these difficulties arise from scanning errors that result in parts of the image, including this fiducial mark, being cut off. Not enough to not find the subset, but so much that the fiducial mark is missing. Figure 11, example A, illustrates such a case. In other rare instances, low contrast between the fiducial pattern and its surroundings complicates detection, as demonstrated by example B in the same figure. In such scenarios, even human observers struggle to pinpoint the fiducial pattern.

When fiducial marks are successfully detected, their positions are generally accurate to within a few pixels, demonstrating strong performance for correctly scanned images. However, errors can occur when a fiducial mark is missing due to scanning issues, or when incorrect scanning introduces artifacts, such as additional fiducial marks from adjacent images, because multiple images were scanned together.

To further evaluate the performance of our fiducial mark extraction, we compared it against the built-in detection algorithm of Agisoft Metashape (see Table 2). Metashape successfully recovers nearly all clearly visible markers and slightly outperforms our method in three of the four cardinal directions. In contrast, our approach, augmented with estimation, matches or exceeds the performance of Metashape for fiducial mark 7 and remains within a few percent for the others. Both methods fail similarly when marks are truncated by scanning errors (e.g., in the case of mark 7). Overall, although Metashape achieves slightly higher detection rates on clean scans, our open-source, fully automated pipeline delivers competitive performance.

**Table 2:** Comparison of fiducial mark detection results between our method and Agisoft Metashape. The table lists the total number of images, the number of images where the fiducial mark is visible, and the number of fid marks by each method. For Fiducial Mark 7, our method detects more instances than the number of visible marks due to its estimation mechanism, which infers likely positions based on consistent placements in neighbouring images.

Fid Mark	Total	Visible	Our method	Agisoft
Fid Mark 5	7719	7711	7607	7695
Fid Mark 6	7719	7714	7607	7701
Fid Mark 7	7719	6015	6667	6012
Fid Mark 8	7719	7662	7607	7659

The extraction of altitude data presents the most significant challenge. Successful extractions based on the altimeter occur in only 15% of all cases (1279 images), often hindered by factors such as inverted images, blurry or illegible altimeter readings, altimeters being cut off, or scanning artifacts affecting the relevant areas. To a lesser extent, these issues also impact manual extraction, which failed in most cases due to altimeters cut off. Figure 11, example C highlights one such problematic example. Additionally, some incorrect altitude values are extracted, necessitating further quality control measures. Despite these issues, the successful extractions provide enough data to estimate missing altitudes along flight paths. This marks a considerable improvement over the previous lack of altitude data for most images. In the context of structure-from-motion (SfM) workflows and workflows aiming at geo-referencing historical imagery lacking GNSS data, (Craciun and Le Bris 2022; Dahle et al. 2024; Giordano et al. 2018), even approximate altitude information can enhance the reconstruction and localization process by providing a valuable starting point.

The extraction of altitude information directly from text, in contrast, is negligible due to the rarity of altitude references within the text boxes. However, in cases where this information is present, the text extraction process typically identifies it correctly.

## Conclusion

However, some challenges remain, particularly in cases involving image degradation, distortions, or non-standard orientations. A notable limitation is the difficulty in reliably detecting fiducial subsets when images are rotated or of poor quality. While our current implementation relies on dlib for object detection, future work could benefit from more advanced methods such as convolutional neural networks (CNNs), which are more robust to variations in image quality and orientation. Integrating a CNN-based approach may further improve detection accuracy and ensure consistent performance across a wider range of image conditions.

Altimeter readings, likewise, remain sensitive to edge detection quality, with factors like noise, lighting artifacts, or obstructions occasionally impairing pointer identification. While altitude text annotations are rare, this reinforces the value of extracting altimeter readings directly from the image. Even though some image collections may include rough altitude metadata, this is not always reliable or consistently available—especially for historical datasets. In these cases, visual extraction of altimeter data offers a valuable complement to existing metadata sources, especially for SfM workflows.

Our current altimeter reading method is based on tailored image processing and known height ranges. While effective, it could be further strengthened by leveraging methods developed for similar tasks. The challenge of reading multi-pointer altimeters closely parallels the well-studied problem of reading analogue clocks and mechanical gauges. Recent work in these areas has employed both traditional vision techniques and deep learning (Chavan et al. 2022; Yang et al. 2022) with promising results. These strategies could serve as a foundation for improving altimeter reading under more variable conditions.

Finally, the methodology could be extended to extract additional visual metadata from historical images, such as analogue capture times or image numbers. Capture time could support sun angle estimation, while image numbers might help link scans to external catalogues. These additions would further enhance metadata completeness and support more powerful, automated processing of historical aerial image collections.

## Appendix

**Table 3:** Flight paths and the number of successful manual extractions for computer vision based parameters.

Flight Path	Direction	Nr. of images	Altimeter	Fid N	Fid E	Fid S	Fid W
1684	L	110	1	110	110	110	110
	R	110	102	0	110	110	110
	V	110	77	110	110	110	110
1813	L	123	123	123	123	123	123
	R	123	121	0	123	123	123
	V	123	123	123	123	123	123
1816	L	182	118	182	182	182	182
	R	182	179	182	182	182	182
	V	182	161	159	182	169	182
1821	L	191	186	191	191	191	191
	R	191	190	191	191	191	191
	V	191	185	191	191	191	191
1822	L	114	112	114	114	114	114
	R	114	103	114	114	114	114
	V	114	1	1	114	114	114
1824	L	109	10	105	105	106	105
	R	109	40	109	109	109	109
	V	109	2	0	109	109	109
1825	L	142	30	142	142	142	142
	R	142	142	0	142	142	142
	V	142	142	142	142	142	142
1826	L	129	129	129	129	129	129
	R	129	129	0	129	129	129
	V	129	129	123	129	129	129
1827	L	111	0	109	111	111	111
	R	111	78	0	111	111	111
	V	111	90	111	107	111	110
1833	L	156	149	156	156	156	156
	R	156	156	156	156	156	156
	V	156	156	156	156	156	156
1846	L	107	72	107	107	107	107
	R	107	107	107	107	107	107
	V	107	107	107	107	107	107
2073	L	115	115	115	115	115	115
	R	115	115	115	115	115	115
	V	115	69	37	115	91	115
2075	L	107	73	107	107	107	107
	R	107	21	107	107	107	107
	V	107	1	18	107	90	107
2136	L	141	16	141	141	141	141
	R	141	141	0	141	141	141
	V	141	140	141	141	141	141
2137	L	145	46	145	145	145	145
	R	145	144	145	145	145	145
	V	145	70	86	145	145	145
2139	L	108	3	108	108	108	108
	R	108	38	108	108	108	108
	V	108	30	10	108	108	108
2140	L	116	7	116	116	116	116
	R	116	37	116	116	116	116
	V	116	47	116	116	116	116
2141	L	121	121	121	121	121	121
	R	121	121	121	121	121	121
	V	121	20	121	121	121	121
2142	L	108	104	108	108	108	108
	R	108	104	108	108	108	108
	V	108	10	108	108	108	108
2143	L	138	138	138	138	138	138
	R	138	62	138	138	138	138
	V	138	1	138	138	138	138

## Footnotes

1 Flight paths with the following id: 1684, 1813, 1816, 1821, 1822, 1824, 1825, 1826, 1827, 1833, 1846, 2073, 2075, 2136, 2137, 2139, 2140, 2141, 2142, 2143.
